# Thyroid Hormone Induces Ca^2+^-Mediated Mitochondrial Activation in Brown Adipocytes

**DOI:** 10.3390/ijms22168640

**Published:** 2021-08-11

**Authors:** Minh-Hanh Thi Nguyen, Dat Da Ly, Nhung Thi Nguyen, Xu-Feng Qi, Hyon-Seung Yi, Minho Shong, Seung-Kuy Cha, Sangkyu Park, Kyu-Sang Park

**Affiliations:** 1 Mitohormesis Research Center, Yonsei University Wonju College of Medicine, Wonju 26426, Gangwon-do, Korea; minh-hanh.tnguyen@yonsei.ac.kr (M.-H.T.N.); dadat.p10@gmail.com (D.D.L.); nguyennhunga2@gmail.com (N.T.N.); skcha@yonsei.ac.kr (S.-K.C.); 2 Department of Physiology, Yonsei University Wonju College of Medicine, Wonju 26426, Gangwon-do, Korea; 3 Department of Global Medical Science, Yonsei University Wonju College of Medicine, Wonju 26426, Gangwon-do, Korea; 4 Key Laboratory of Regenerative Medicine, Ministry of Education, Department of Developmental and Regenerative Biology, Jinan University, Guangzhou 510632, China; qixufeng@jnu.edu.cn; 5 Department of Internal Medicine, Chungnam National University School of Medicine, Daejeon 35015, Korea; jmpbooks00@gmail.com (H.-S.Y.); minhos@cnu.ac.kr (M.S.); 6 Department of Precision Medicine, Yonsei University Wonju College of Medicine, Wonju 26426, Gangwon-do, Korea

**Keywords:** brown adipose tissue (BAT), thyroid hormone, mitochondria, uncoupling protein 1 (UCP1), Ca^2+^ signaling

## Abstract

Thyroid hormones, including 3,5,3′-triiodothyronine (T_3_), cause a wide spectrum of genomic effects on cellular metabolism and bioenergetic regulation in various tissues. The non-genomic actions of T_3_ have been reported but are not yet completely understood. Acute T_3_ treatment significantly enhanced basal, maximal, ATP-linked, and proton-leak oxygen consumption rates (OCRs) of primary differentiated mouse brown adipocytes accompanied with increased protein abundances of uncoupling protein 1 (UCP1) and mitochondrial Ca^2+^ uniporter (MCU). T_3_ treatment depolarized the resting mitochondrial membrane potential (Ψ_m_) but augmented oligomycin-induced hyperpolarization in brown adipocytes. Protein kinase B (AKT) and mammalian target of rapamycin (mTOR) were activated by T_3_, leading to the inhibition of autophagic degradation. Rapamycin, as an mTOR inhibitor, blocked T_3_-induced autophagic suppression and UCP1 upregulation. T_3_ increases intracellular Ca^2+^ concentration ([Ca^2+^]_i_) in brown adipocytes. Most of the T_3_ effects, including mTOR activation, UCP1 upregulation, and OCR increase, were abrogated by intracellular Ca^2+^ chelation with BAPTA-AM. Calmodulin inhibition with W7 or knockdown of MCU dampened T_3_-induced mitochondrial activation. Furthermore, edelfosine, a phospholipase C (PLC) inhibitor, prevented T_3_ from acting on [Ca^2+^]_i_, UCP1 abundance, Ψ_m_, and OCR. We suggest that short-term exposure of T_3_ induces UCP1 upregulation and mitochondrial activation due to PLC-mediated [Ca^2+^]_i_ elevation in brown adipocytes.

## 1. Introduction

Adipose tissues are traditionally divided into two types: brown adipose tissue (BAT) and white adipose tissue (WAT), based on the differences in stem cell origin, anatomy, morphology, cell structure, and function [1]. While the main function of white fat is energy storage in the form of a single intracellular lipid droplet (unilocularity), brown adipocytes, which are multilocular, dissipate energy via heat production by proton currents through uncoupling protein 1 (UCP1) [2]. 

UCP1 is highly expressed in BAT. This channel is located on the mitochondrial inner membrane and allows protons to translocate from the intermembrane space to the matrix. The proton currents via UCP1 dissipate energy as heat production, which is uncoupled with substrate oxidation, electron transport chain (ETC) activities, and ATP production. In BAT, UCP1 abundance accounts for about 10% of the mitochondrial membrane protein, while UCP2 and UCP3 constitute only about 0.01~0.1% [3]. Energy dissipation via UCP1 of BAT potentially enhances the total energy expenditure of the whole body up to 20% [4]; therefore, UCP1 activation is considered a potentially promising therapeutic approach for obesity and diabetes treatment [5]. Although proton leak through UCPs is responsible for most of the uncoupling respiration in mitochondria, which generates heat instead of ATP, the heat production is regulated by the rate of mitochondrial respiration as a whole and not by UCPs alone. The elevation of free energy loss as heat generation can happen through the modification of several variables, such as increasing ATP consumption, UCP activities, futile ATP hydrolysis, and reducing electron transfer efficacy [6].

Thyroid hormones (TH), including thyroxine (T_4_) and its more active form 3,5,3′-triiodothyronine (T_3_), have been widely accepted and applied in clinical practices because of their wide spectrum of effects on the regulation of cellular metabolism, cell structure, and membrane transport [7]. These effects are traditionally thought to depend on the transcriptional modulation of specific genes after binding on the hormonal responsive elements in promoters by intranuclear complexes of TH and thyroid receptor (TR) as homodimers or, most popularly, as heterodimers with retinoid X receptor (RXR) [7]. Before binding to TR in the nucleus, TH is required to enter the cells by carrier-mediated transport and diffusion [8]. The binding of TH-TR complexes to target genes increases the expression of either functional protein, causing alterations in cell metabolism, or structural proteins, which are responsible for cell growth. This process usually takes time, from several hours to several days.

The non-genomic actions of TH, which are independent of nuclear translocation and TH-TR complex formation, have been reported in the past few decades, but their molecular mechanisms remain unclear [7,9]. Generally, if a particular biological effect is considered to be mediated by a non-genomic pathway, it should meet the following criteria: (1) The action is rapid in onset (minutes or a few hours) in comparison to transcription or translation, (2) Gene transcription and protein synthesis are not required for the action [9]. Non-genomic actions of TH can be initiated by binding to receptors located on the cytosolic membrane, cytoplasm, or mitochondria [9]. For example, TH activates PI3K or MAPK when binding to integrin αvβ3 receptor at S1 or S2 domain, respectively [10,11]; TH promotes actin polymerization within 20 min possibly through cytoplasmic truncated isoforms of TRα1 and TRα2 [12]. Two N-terminal truncated forms of TRα1, p43 and p28, were also detected on mitochondrial extracts of rat liver [13]. It should be noted that over a long period of time, an initial non-transcriptional alteration in the cellular signaling pathway may initiate changes in gene expression, which results in the overlap of genomic and non-genomic effects. However, in this context, the changes may result in the alterations of several aspects of cellular functions rather than a single gene activation. Therefore, the term ‘non-genomic’ should be understood as the effects that do not require direct binding of TH (or TH-TR complexes) to gene promoters. 

Until now, most of the activities of TH in BAT have been reported through genomic pathways requiring a relatively long time to perform the biological changes. In particular, T_3_ regulates a variety of genes (i.e., *Cebp*s, *Ppar*s, and *Ppargc1a*) associated with the differentiation of adipose tissue, which involves lipogenesis, lipolysis, and thermogenesis in BAT [14]. T_3_ has also been demonstrated to enhance the adrenergic stimulation of UCP1 [15]. This hormone can directly upregulate UCP1 expression in the primary cells of fetal rats [16]. Besides, TH plays an important role in facultative thermogenesis by activating the expression of the genes involved in lipid mobilization and β-oxidation, which contributes materials for proton gradient generation and activates UCP1 activity by the direct binding of long-chain FFA to the pore region of UCP1 [17].

Here, based on the aforementioned results obtained by applying T_3_ treatment for 30 min, we propose a novel molecular mechanism of T_3_ for enhancing mitochondria activities in brown adipocytes in a transcription-independent manner. 

## 2. Results

### 2.1. Acute T_3_ Exposure Induces Mitochondrial Activation in Brown Adipocytes 

#### 2.1.1. T_3_ Increases Mitochondrial Respiration 

**Figure 1:**
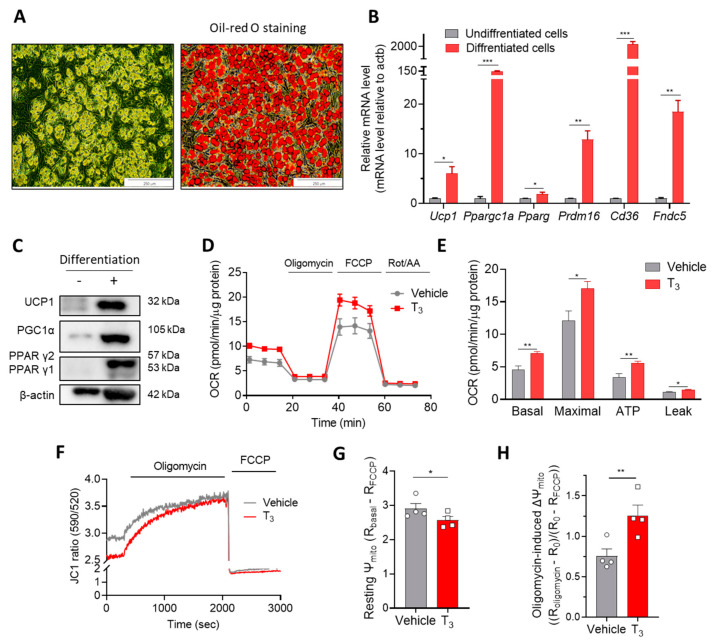
T_3_ induces mitochondrial activation in brown adipocytes within 30 min. (**A**) Oil red O staining of differentiated primary mouse brown adipocytes. (**B**) Relative mRNA levels of *Ucp1, Ppargc1a, Pparg, Cd36, Fndc5* (normalized to *Actb*) (*n* = 3) and (**C**) western blot of UCP1, PGC1α and PPARγ of undifferentiated and differentiated primary brown adipocytes. (**D**) OCR measurement in primary brown adipocytes with 10 nM T_3_ for 30 min. (**E**) Quantitative analysis of basal respiration, maximal respiration, ATP production, and proton leak (*n* = 4). (**F**) Mitochondrial membrane potential (Ψm) measurement with JC1 dye on immortalized brown adipocytes with 10 nM T_3_ treatment for 30 min. (**G**) Quantitative analysis of resting Ψm and (**H**) Oligomycin-induced hyperpolarization (∆Ψm, *n* = 4). Data are presented as mean ± SEM. * *p* ≤ 0.05, ** *p* ≤ 0.01, *** *p* < 0.001.

Differentiated (mature) mouse primary and immortalized brown adipocytes were used for most of the experiments in this study. Adipocytes started to accumulate lipids on day four (after the induction phase) and were tightly compressed with lipid droplets after 10 days of differentiation (Figure S1A). The differentiation efficiency was estimated by visualizing lipid accumulation with oil red O staining (Figure 1A and Figure S1B) or quantifying mRNA and protein levels of differentiation markers (Figure 1B,C and Figure S1C,D). The transcriptional levels of differentiation markers of brown adipocytes (*Ucp1*, *Ppargc1a*, *Pparg*) and regulators of lipid uptake (*Cd36*) and thermogenesis (*Fndc5*) were significantly increased in the differentiated adipocytes (Figure 1B and Figure S1C). The protein levels of UCP1, PGC1α, and PPARγ were also markedly upregulated on days 10–14 of differentiation (Figure 1C and Figure S1D). 

Treatment of T_3_ for 24 h enhanced the oxygen consumption rate (OCR) in a variety of cell types [18,19]. The mitochondria isolated from mouse livers at 30 min after intravenous T_3_ injections also showed higher OCR and ATP production compared to those of mitochondria from control livers [20]. To investigate whether acute T_3_ exposure can activate mitochondrial activities, brown adipocytes were treated with different concentrations of T_3_ ranging from 5 nM to 100 nM for 30 min. T_3_ treatment increased mitochondrial respiration of immortalized brown adipocytes, achieving the highest activation at 10 nM (Figure S1E,F). In primary brown adipocytes, T_3_ (10 nM) increased all parameters of mitochondrial activities, including basal, maximal, proton leak, and ATP-linked OCRs (Figure 1D,E). Recent studies suggest the OCR measurement using an activator of lipolysis and bovine serum albumin (BSA) in order to estimate the capacity of UCP1-dependent proton leak [21].

#### 2.1.2. T_3_ Alters Mitochondrial Membrane Potential (Ψm) 

The activities of ETC, ATP synthase, and proton leak channels are critical components for the development and regulation of Ψm because all of them can affect the proton gradient across the inner membrane. To determine whether the changes in OCR with T_3_ treatment could alter the Ψm of brown adipocytes, the fluorescence intensities of JC1 dye were measured with or without 30 min treatment with T_3_. The ratio of two emission wavelengths, red/green (590 nm/535 nm), proportionally reflects the ΔΨm (Figure 1F). T_3_ treatment induced the depolarization of resting Ψm (Figure 1G). Interestingly, however, oligomycin-induced hyperpolarization of the Ψm was enhanced by T_3_ treatment (Figure 1H). The depolarized resting Ψm and augmented oligomycin response by T_3_ may result from increased proton leak and ATP synthase activity, respectively.

### 2.2. Changes in Cellular Function by Acute T_3_ Treatment 

#### 2.2.1. T_3_ Suppresses Autophagic Degradation

**Figure 2:**
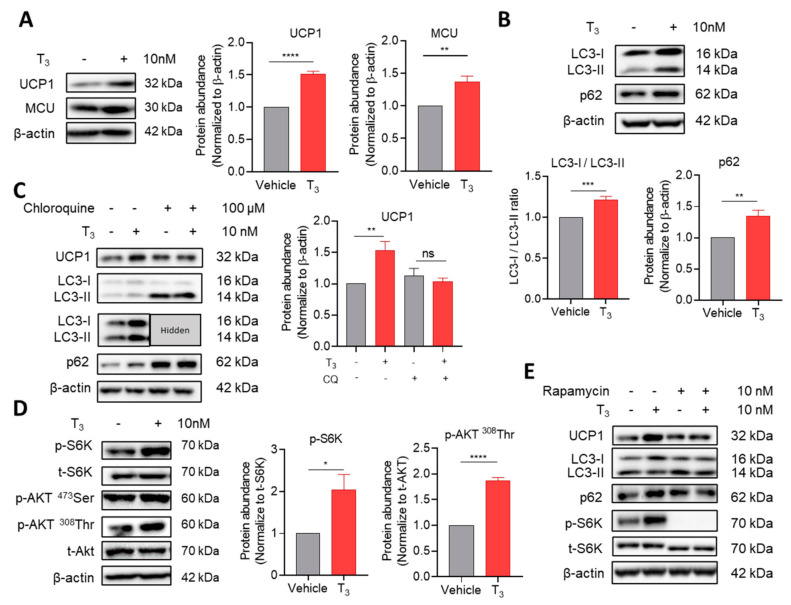
T_3_ inhibits autophagic degradation by mTOR activation. (**A**) Western blot and quantitative analysis of UCP1 (*n* = 5) and MCU (*n* = 4) with 10 nM T_3_ treatment for 30 min in immortalized brown adipocytes. (**B**) Western blot of autophagy-related proteins with 10 nM T_3_ treatment for 30 min in immortalized brown adipocytes and quantitative analysis of LC3 I/LC3 II (*n* = 6), and p62 (*n* = 5). (**C**) Western blot of UCP1 and autophagy-related proteins after preincubation with 100 μM chloroquine for 24 h prior to T_3_ treatment and quantitative analysis of UCP1 (*n* = 9). (**D**) Western blot and quantitative analysis of mTOR signaling proteins under T_3_ treatment (*n* = 4). (**E**) Western blot of UCP1, autophagy-related proteins, and S6K under preincubation with 10 nM rapamycin for 1 h prior to T_3_ treatment. Data are presented as mean ± SEM. * *p* ≤ 0.05, ** *p* ≤ 0.01, *** *p* < 0.001, **** *p* < 0.0001, ns not significant.

The attenuation of autophagy induces browning and increased thermogenesis, while the acceleration of autophagy may decrease the mitochondrial protein amount and hasten the whitening of adipose tissue [22]. In our model, T_3_ treatment for 30 min increased the abundance of mitochondrial proteins, including UCP1 and mitochondrial calcium uniporter (MCU) (Figure 2A and Figure S2A). Besides, LC3, a marker for autophagosomes, was upregulated, and the ratio of LC3-I/LC3-II was increased (Figure 2B and Figure S2A). The accumulation of an autophagy substrate, p62, was observed with T_3_ treatment (Figure 2B and Figure S2A). Furthermore, the effects of T_3_ on LC3, p62, and UCP1 were abrogated by pretreatment with chloroquine, which inhibits lysosomal function (Figure 2C), suggesting that T_3_ suppresses autophagolysosomal degradation [23]. Notably, T_3_ treatment for 30 min did not cause any changes in the transcriptional levels of *Ucp1* or autophagy markers (*Map1lc3a*, *Map1lc3b, Rps6*) in immortalized or primary brown adipocytes (Figure S2C,D). 

#### 2.2.2. T_3_-Induced Mammalian Target of Rapamycin (mTOR) Activation Inhibits Autophagic Degradation

The Western blot data on immortalized or isolated mouse primary brown adipocytes showed that T_3_ treatment for 30 min increased the phosphorylation of ribosome protein S6 kinase (S6K) as a downstream of mTOR activation (Figure 2D and Figure S2B) [24]. T_3_ activated protein kinase B (AKT) as an upstream regulator of mTOR signaling by phosphorylating the ^308^Thr residue but not at ^473^Ser residue (Figure 2D) [25]. To demonstrate whether autophagy suppression is caused by mTOR activation [26], adipocytes were treated with 10 nM rapamycin, an mTOR inhibitor, for an hour before T_3_ application, which resulted in the complete suppression of S6K phosphorylation (Figure 2E). Under preincubation with rapamycin, the effects of T_3_ on autophagy-related proteins (LC3 and p62) and UCP1 abundance were abolished (Figure 2E). These results indicate that increasing UCP1 abundance by T_3_ could have resulted from mTOR-mediated inhibition of autophagic degradation.

### 2.3. T_3_ Induces Cytosolic Ca^2+^ Increase and Mitochondrial Activation

#### 2.3.1. T_3_ Induces a Rapid and Sustained [Ca^2+^]_i_ Elevation 

**Figure 3:**
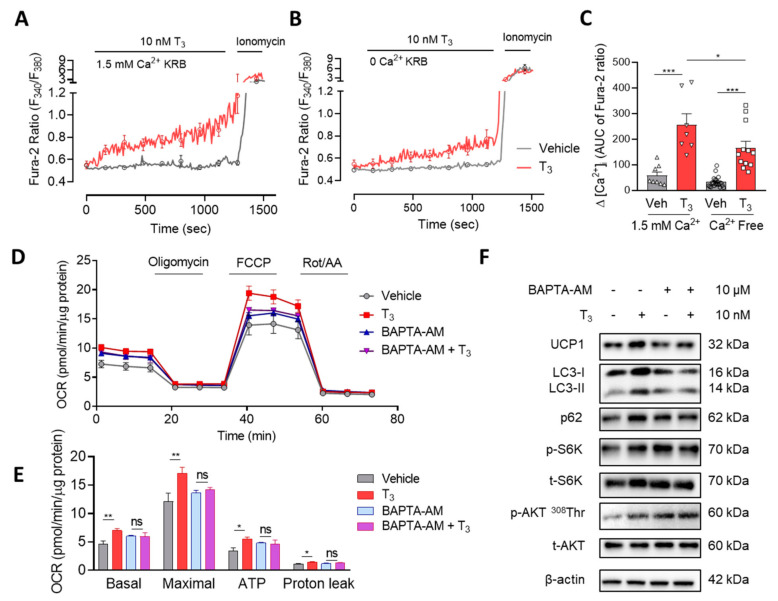
T_3_ induces cytosolic Ca^2+^ increase and mitochondrial activation. Cytosolic Ca^2+^ measurement with Fura-2 AM in (**A**) free Ca^2+^ and (**B**) 1.5 mM Ca^2+^ KRB on immortalized brown adipocytes with direct 10 nM T_3_ perfusion. (**C**) Quantitative analysis of [Ca^2+^]_i_ in free and 1.5 mM Ca^2+^ KRB. (**D**) OCR measurement on primary brown adipocytes with 10 μM BAPTA-AM treatment for 1 h before 10 nM T_3_ treatment for 30 min. (**E**) Quantitative analysis of basal respiration, maximal respiration, ATP production and proton leak (*n* = 4). (**F**) Western blot of several autophagy-related proteins and mTOR signaling pathway proteins in immortalized brown adipocytes with 10 μM BAPTA-AM pretreatment for 1 h before 10 nM T_3_ treatment for 30 min. Data were presented as mean ± SEM. * *p* ≤ 0.05, ** *p* ≤ 0.01, *** *p* < 0.001, ns not significant.

To examine the non-genomic regulation of T_3_ on cytosolic Ca^2+^ signaling, the brown adipocytes were loaded with Fura-2 AM for 40 min before measurement with a live-cell system. The ratio of two emission wavelengths, 340 nm/380 nm, proportionally reflects the changes of cytosolic Ca^2+^ level ([Ca^2+^]_i_). T_3_ induced a rapid and sustained [Ca^2+^]_i_ increase in a Krebs–Ringer bicarbonate (KRB) solution containing 1.5 mM Ca^2+^. The response was attenuated but maintained in a Ca^2+^-free KRB (Figure 3A–C). These results suggest that elevated [Ca^2+^]_i_ originates from intracellular Ca^2+^ stores as well as the extracellular environment.

#### 2.3.2. T_3_ Activates Mitochondrial Respiration via Ca^2+^ Signaling

To investigate the relationship between [Ca^2+^]_i_ elevation and OCR increase by T_3_, brown adipocytes were pretreated with BAPTA-AM, an intracellular Ca^2+^ chelator, 1 h before 10 nM T_3_ treatment and mitochondrial respiration was measured. Cytosolic Ca^2+^ depletion completely suppressed T_3_-induced enhanced effects on basal, maximal, ATP-linked, and proton leak OCR in both immortalized and primary brown adipocytes (Figure 3D,E and Figure S3A,B). These results provide evidence for the critical role of cytosolic Ca^2+^ elevation in T_3_-induced mitochondrial activation. 

Moreover, pretreatment with 2-aminoethoxydiphenyl borate (2-APB) also blocked OCR increases by T_3_ (Figure S3C,D). 2-APB acts as a nonselective inhibitor of inositol 1,4,5-triphosphate (IP_3_)-induced ER Ca^2+^ release, transient receptor potential channels (TRPC), and store operated Ca^2+^ entry (SOCE) [27,28]. This evidence reinforced the role of cytosolic Ca^2+^ in T_3_ effects on mitochondrial respiration.

#### 2.3.3. T_3_-Induced mTOR Activation via Ca^2+^ Signaling 

To investigate the role of Ca^2+^ on T_3_-induced mTOR activation, brown adipocytes were preincubated with BAPTA-AM for 1 h, and then the changes in mTOR and autophagic signaling by T_3_ were examined. Cytosolic Ca^2+^ chelation prevented the effects of T_3_ on mTOR activation and inhibition of autophagic degradation in immortalized brown adipocytes (Figure 3F). Pretreatment with 2-APB exhibited similar inhibitory effects on T_3_–induced changes (Figure S3E).

Calmodulin (CaM) binds Ca^2+^ binding protein, which works as a component of the Ca^2+^ signal transduction pathway regulating the activities of a variety of downstream targets, such as protein kinases and phosphatases [29]. To understand whether CaM is involved in Ca^2+^-mediated T_3_ action, immortalized brown adipocytes were pretreated with W7, a CaM inhibitor, for 1 h before T_3_ treatment. W7 reduced the phosphorylation of S6K and AKT at ^308^Thr as well as the attenuation of autophagy degradation, indicating the involvement of CaM in the T_3_ regulation of mTOR, autophagic degradation, and mitochondrial activities (Figure S4B). However, the pretreatment with W7 only partially suppressed T_3_–activated OCR changes (Figure S4A). This suggests the involvement of other factors in the T_3_-induced OCR increase.

#### 2.3.4. Cytosolic and Mitochondrial Ca^2+^ Mediate T_3_-Activated Mitochondrial Respiration 

Mitochondrial Ca^2+^ uptake via MCU activates matrix dehydrogenase and ATP synthase [30]. To demonstrate the role of mitochondrial Ca^2+^ in T_3′_s action, immortalized brown adipocytes were transfected with siRNA specific to *Mcu*. The silencing efficacy was confirmed by estimating MCU protein abundance (Figure S4C). Knockdown of MCU partially inhibited the changes in mitochondrial respiration by T_3_ (Figure S4D). Interestingly, the combination of CaM inhibition and *Mcu* knockdown additively suppressed T_3_-induced OCR increase, which indicates that both mitochondrial Ca^2+^ influx and cytosolic [Ca^2+^]_i_-CaM contribute to the effects of T_3_ on mitochondrial respiration (Figure S4E,F). 

### 2.4. Phospholipase C (PLC) Is Involved in T_3_-Induced [Ca^2+^]_i_ Increase and Mitochondrial Activation

**Figure 4:**
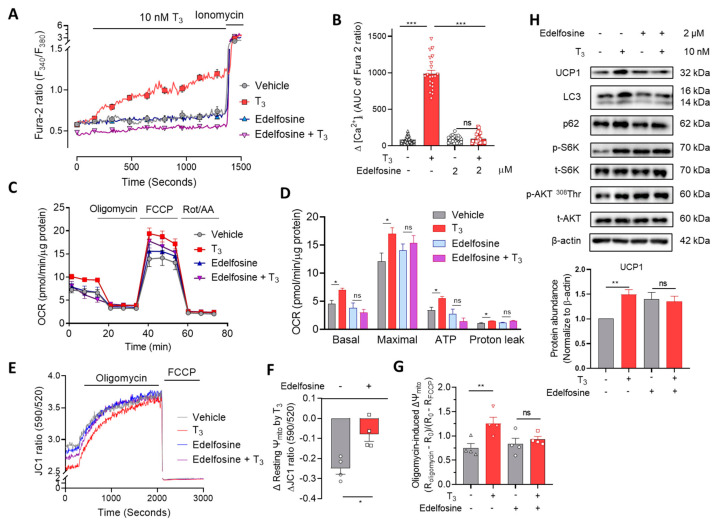
PLC inhibitor prevents T_3_-induced [Ca^2+^]_i_ increase and mitochondrial activation. (**A**) Cytosolic Ca^2+^ measurement by Fura-2 AM with or without 2 μM edelfosine treatment for 6 h before 10 nM T_3_ perfusion on immortalized brown adipocytes. (**B**) Quantitative analysis of cytosolic Ca^2+^ in vehicle- or T_3_-treated group with and without edelfosine treatment. (**C**) OCR measurement in primary brown adipocytes with 2 μM edelfosine treatment for 6 h before 10 nM T_3_ treatment for 30 min. (**D**) Quantitative analysis of basal respiration, maximal respiration, ATP production, and proton leak (*n* ≥ 5) (**E**) Ψm measurement using JC1 dye with 10 nM T_3_ treatment for 30 min in immortalized brown adipocytes. (**F**) Quantitative analysis of T_3_-induced differences in resting Ψm with and without edelfosine treatment. (**G**) Quantitative analysis of oligomycin-induced hyperpolarization. (**H**) Western blot of UCP1, autophagy-related proteins, and mTOR signaling proteins with 2 μM edelfosine treatment for 6 h before 10 nM T_3_ treatment in immortalized brown adipocytes and quantitative analysis of UCP1. Data were presented as mean ± SEM. * *p* ≤ 0.05, ** *p* ≤ 0.01, *** *p* < 0.001, ns not significant.

Phospholipase C (PLC) resides on the plasma membrane and selectively catalyzes the hydrolysis of phosphatidylinositol 4,5-bisphosphate (PIP_2_) into diacylglycerol (DAG) and inositol 1,4,5-triphosphate (IP_3_) [31]. T_3_ increased the activity of PLC on immortalized undifferentiated cells, which was blunted by edelfosine (PLC inhibitor) pretreatment (Figure S5A). Because of the technical problem, these results were obtained from undifferentiated cells, which may differ from mature adipocytes. Treatment of immortalized brown adipocytes with 2 μM edelfosine prevented the T_3_-induced [Ca^2+^]_i_ elevation (Figure 4A,B). In addition to the T_3_-induced OCR increase, mitochondrial membrane potential changes were also blocked by edelfosine pretreatment (Figure 4C–G and Figure S5C). Consistently, mTOR activation and autophagic degradation inhibition were also suppressed by treatment with either edelfosine (Figure 4H and Figure S5D) or U73122—another PLC inhibitor (Figure S5E). These data suggest that T_3_ induced [Ca^2+^]_i_ elevation by regulating PLC, which in turn activated mitochondrial respiration either directly or indirectly via CaM activation. 

## 3. Discussion

Although thyroid hormones have been demonstrated to have numerous positive effects on thermogenesis in brown adipocytes, most of them occur through genomic actions [14,15,16]. Our findings point out that T_3_ induces mitochondrial activation within 30 min, which is dependent on [Ca^2+^]_i_ elevation. It has been reported that T_3_ increases [Ca^2+^]_i_ within several minutes in myoblast cell lines and thyroid hormone receptor βA1-expressing oocytes [32,33,34]. Although IP_3_ was reported to participate in T_3_-induced [Ca^2+^]_i_ increase, there has not been any direct evidence demonstrating the involvement of PLC as a downstream signal of non-genomic T_3′_s action [32,34]. Here, we demonstrated that PLC mediates acute effects of T_3_ on mitochondrial activation in brown adipocytes. 

Acute treatment of T_3_ increases mitochondrial respiration, probably as a result of multiple changes in the cells that are intertwined with each other. We found that the alterations in autophagy-regulating signals associated with [Ca^2+^]_i_-bound CaM led to the upregulation of mitochondrial protein abundance. Additionally, direct stimulation of mitochondrial metabolism by MCU-mediated mitochondrial Ca^2+^ uptake also participated in the activation of mitochondrial respiration. These two signals originated from T_3_–induced [Ca^2+^]_i_ elevation due to PLC-mediated intracellular Ca^2+^ release and extracellular Ca^2+^ entry.

The exposure of isolated skeletal muscle mitochondria to high Ca^2+^ solution activates the entire mitochondrial oxidative phosphorylation pathway in a dose-dependent manner. In particular, at the optimal concentration of Ca^2+^ (840 nM), the conduction of complex IV increases 2.3-fold, complexes I and III 2.2-fold, and ATP synthase is 2.4-fold [35]. Our data on OCR measurement shows that T_3_-induced high [Ca^2+^]_i_ activates not only the electron transport chain but also ATP production. However, the increase in the proton leak current in brown adipocytes may occur as a secondary consequence of increased UCP1 abundance and the phosphorylation of AMPK and its downstream acetyl-CoA carboxylase (ACC) [25,26,36] (Figure S6). ACC catalyzes the carboxylation of acetyl-CoA to malonyl-CoA, the first step in fatty acid synthesis. The inhibition of ACC by phosphorylation was demonstrated by previous studies to enhance the acetyl-CoA level, which then provides acetyl groups for Krebs cycle to generate reducing agents donating electrons for ETC activities [37]. Furthermore, Lee et al. showed that the phosphorylated ACC also facilitates long-chain FFA uptake into mitochondria and increases proton leak through FFA binding to UCP1 [38]. 

One of the interesting findings is that both mTOR signaling and AMPK signaling are activated by acute T_3_ treatment. In autophagy regulation, mTOR and AMPK play opposite roles; mTOR inhibits and AMPK activates autophagic degradation [39,40,41]. We suppose that the Ca^2+^-CaM complex could be responsible for the activation of the signaling pathways. For the activation process of mTOR by sensing amino acids, Ca^2+^/CaM-dependent protein kinase (CaMK) is required [42,43]. Furthermore, CaMK also activates AKT, which consequently leads to mTOR activation [44]. We observed in this study that T_3_ enhances phosphorylation at ^308^Thr residue of AKT, which contributes to mTOR activation. Activation of mTOR can suppress the lysosomal Trp-ML1 channel, and Ca^2+^ release from this channel is critical for autophagosome-lysosome fusion and degradation [45]. We suggest that the inhibition of autophagic degradation could contribute to the accumulation of mitochondrial proteins involved in respiratory activity.

According to the OCR measurement results, T_3_ enhanced the activity of ETC, which transports protons from the mitochondrial matrix into the intermembrane space. T_3_ also increased ATP synthase and proton leak channels, both of which translocate protons into the matrix allowing the collapse of the electrochemical gradient. These bidirectional proton transports determine the mitochondrial electrical gradient (Ψm) in a complicated way. Increased inward proton leak can attenuate the electrochemical gradient and depolarize the resting Ψm. On the contrary, augmented proton influx via ATP synthase can be detected as heightened hyperpolarizing responses to block this transport by oligomycin. T_3_ showed depolarization of the resting Ψm and exaggerated hyperpolarizing changes to oligomycin treatment. However, all these changes are possible only if there is a robust electrochemical gradient driven by increased ETC activities. T_3_ increased MCU protein abundance, and induced cytosolic Ca^2+^ elevation could allow an increase in mitochondrial matrix Ca^2+^ uptake via MCU. Therefore, T_3_-induced Ψm alterations were abrogated by edelfosine-mediated inhibition of [Ca^2+^]_i_ changes and mitochondrial activities.

In summary, we identify a novel molecular mechanism by which the short-term treatment of T_3_ increases mitochondrial protein abundance and activates mitochondrial respiration in a PLC-mediated [Ca^2+^]_i_ elevation-dependent manner in brown adipocytes. This Ca^2+^ signaling regulates not only mitochondrial metabolism via MCU-mediated Ca^2+^ uptake but also autophagic protein degradation leading to the accumulation of mitochondrial proteins, possibly contributing to acute thermogenic stimulation. Identifying the binding partners of T_3_ to induce these activations could be an effective therapeutic strategy for treating metabolic and age-related diseases [46,47]. 

## 4. Materials and Methods

### 4.1. Reagents

3,5,3′-triiodothyronine (catalog no. T2877), BAPTA-AM (catalog no. A1076), antimycin A (catalog no. A8674), edelfosine (catalog no. sml0332), and chloroquine phosphate (catalog no. PHR1258) were purchased from Sigma-Aldrich (St. Louis, MO, USA).

### 4.2. Immortalized Cell Culture

Immortalized pre-mature brown adipocytes were cultured in DMEM-F12 medium (catalog no. 11330032, Gibco, Waltham, MA, USA) with 10% FBS (catalog. 16000044, Gibco) and 1% penicillin/streptomycin (P/S; catalog no. SV30010, Hyclone, Logan, UT, USA) to achieve a cell confluency of about 70% and then, differentiated in two phases: induction (4 days) and maintenance (10–12 days) before experiments. In the first phase, the cells were incubated in the regular medium (including 10% FBS, 1% P/S) supplemented with 10 μg/mL insulin (catalog no. I2643, Sigma-Aldrich, St. Louis, MO, USA), 10 μM rosiglitazone (catalog no. R2408, Sigma-Aldrich, St. Louis, MO, USA), 125 μM indomethacin (catalog no. I7378, Sigma-Aldrich, St. Louis, MO, USA), and 500 μM 3-isobutyl-1-methaxine (IBMX). The maintenance medium was regular medium (with 10% FBS, 1% P/S) added with 10 μg/mL insulin. The differentiation efficiency was estimated by testing lipid accumulation using oil red O staining and brown adipocyte markers using qPCR and Western blotting. 

### 4.3. Primary Cell Isolation

All protocols for animal care and procedures have been approved by the Yonsei University Wonju College of Medicine Institutional Animal Care and Use Committee. The approval number is YWC-201023-1. 

Primary brown adipocytes were isolated from 6~8-week old female C57BL/6 mice, referring to the protocol reported previously [48]. The brown adipose tissue was then minced into small pieces and dissected in a dissection medium. The dissection medium was prepared by adding 1.5 μ/mL collagenase D (catalog no. 1108874103, Roche, Penzberg, Germany) and 2.4 μ/mL Dispase II (catalog no. 04942078001, Roche, Penzberg, Germany) in PBS solution. The medium was supplemented with 10 mM CaCl_2_ right before digestion to activate the enzymes. BAT was digested with stable agitation at 150 rpm for 40~50 min at 37 °C. The dissection was stopped by adding complete medium (DMEM/F12 containing 10% FBS and 1% P/S). After centrifuging at 700× *g* for 10 min and removing the oily and liquid layers on the top, a brownish pellet was collected at the bottom of the tube and then re-dissolved in complete medium. The cell suspension was filtered with a cell strainer (50 μm diameter) and centrifuged at 700× *g* for 10 min. The cell pellet was resuspended in complete medium and seeded on collagen-coated dishes. The cells were washed with PBS twice, and fresh medium was introduced after 3 h.

After the primary brown adipocytes achieved about 70% of confluency, they were differentiated into mature brown adipocytes by following the same protocol as that of the immortalized cells.

### 4.4. Oxygen Consumption Rate (OCR) Measurement

OCR was measured with an Extracellular Flux Analyzer XF-96 (Agilent, Santa Clara, CA, USA). The immortalized and primary brown adipocytes were seeded at a density of 30,000 cells/well and 20,000 cells/well, respectively, in a Seahorse 96-well plate that was coated with poly L-lysine or collagen a day before the experiment. The cells were incubated in XF DMEM pH = 7.4 (catalog no. 103575-100, Agilent, Santa Clara, CA, USA) along with 1 mM pyruvate, 2 mM glutamine, and 17.5 mM glucose an hour before the measurement. The basal OCR and the OCR after injections of drugs, including 5 uM oligomycin (catalog no. A 75351, Sigma-Aldrich, St. Louis, MO, USA), 5 μM carbonyl cyanide 4-(trifluoromethoxy) phenylhydrazone (C2920, Sigma-Aldrich, St. Louis, MO, USA), 2 μM rotenone (catalog no. R8755, Sigma), and 2 μM antimycin A, were measured every 18 min (including 3 cycles of mixing and measuring alternately).

### 4.5. Calcium Measurement by Live-Cell Imaging

The immortalized cells were seeded on poly-l-lysine-coated 12 mm coverslips at a density of 30,000 cells/coverslip a day before the experiment. The cells were incubated with 5 μM Fura-2 AM (catalog no. F1201, Invitrogen, Waltham, MA, USA) dissolved in Krebs–Ringer-bicarbonate (KRB) solution (containing 135 mM NaCl, 5.4 mM KCl, 1 mM MgCl_2_, 2 mM CaCl_2_, 5 mM HEPES, and 5.5 mM glucose) for 40 min in the dark at 37 °C. After that, the coverslip was transferred to a perfusion chamber, installed on an inverted microscope, and alternately excited at the wavelengths of 340 and 380 nm by a monochromatic light source (Lamda DG-4, Sutter Instruments, Novato, CA, USA). The chamber was perfused with KRB (without or with treatment). Emission signals were recorded at a wavelength of 510 nm by an intensified CCD camera (Cascade, Roper, Duluth, GA, USA) and the ratio of fluorescence intensities (F340/F380) reflecting [Ca^2+^]_i_ was analyzed with MetaFluor 6.1 software (Molecular Devices, Sunnyvale, CA, USA). At the end of each measurement, 10 μM ionomycin dissolved in KRB was perfused as a positive control.

### 4.6. SDS-PAGE/Western Blotting

The cells were washed with cold PBS on ice and lysed with cold Pro-prep buffer (catalog no. 17081, Intron Biotechnology, Gyeonggi-do, Korea) supplemented with phosphatase and protease inhibitors (catalog no. 4906837001 and 5892791001, Sigma-Aldrich, St. Louis, MO, USA respectively). Lysates were centrifuged at 15,000 rpm for 20 min at 4 °C to collect supernatants. Protein concentration was measured using a BCA protein assay kit (catalog no. 23225, Thermo Fisher Scientific, Waltham, MA, USA) before being electrophoresed on SDS-PAGE gels with an equal amount of protein in each well. The proteins were then electroblotted onto a polyvinylidene difluoride membrane (Merck Millipore, Billerica, MA, USA). The membrane was blocked in 6% skim milk for 1 h at room temperature before being incubated with a primary antibody at 4 °C for 18 h. The following primary antibodies were used: p-p70S6K (Thr389) (catalog. no 9205), p70S6K (catalog. no 9202), p-ACC (Ser79) (catalog no. 3661), ACC (catalog no. 3662), p-AKT (^473^Ser) (catalog no. 9271), p-AKT (^308^Thr) (catalog no. 9275), AKT (catalog no. 9272), and PPARγ (catalog no. 2443) from Cell Signaling Technology (Danvers, MA, USA) and β-actin (catalog no. ab6276) and PGC1α (catalog no. ab54481) from Abcam (Cambridge, UK). All primary antibodies were diluted to 1:1000 in 5% BSA dissolved in a solution of 0.1% Tris-buffered saline and Tween 20 (TBST). The membranes were then incubated with horseradish peroxidase-conjugated secondary antibody against mouse or rabbit IgG (catalog no. 31460 or 31450 respectively, Thermo Fisher Scientific, Waltham, MA, USA) diluted in 6% skim milk in TBST for 1 h at room temperature (23 °C). Immunoreactive bands were detected by using ECL solution (Luminata Fort, Millipore Corp., Burlington, MA, USA, catalog no. WBLUF0100) and Chemi Doc XRS+ imaging system and were analyzed using Image Lab software 6.0 (Bio-Rad, Hercules, CA, USA).

### 4.7. RNA Extraction, cDNA Synthesis, and Quantitative Real-Time PCR

Brown adipocytes were lysed by RiboEx (catalog no. 301-902, GeneAll Biotechnology, Seoul, Korea). The lysates were mixed with chloroform and centrifuged to collect the upper aqueous phase containing mRNA. A rough mRNA pellet was collected by centrifuging the mixture of this aqueous phase with isopropyl alcohol after incubating at −80 °C for 1 h. The mRNA was roughly washed with 75% ethanol to purify and re-dissolved in 0.1% diethylpyrocarbonate (DEPC)-treated water. Complementary DNA was synthesized from total RNA with ReverTra Ace^TM^ qPCR RT Master Mix and gDNA remover (catalog no. FSQ-301, Toyobo, Osaka, Japan) by following the manufacture’s protocol.

The mRNA levels of brown adipocytes were checked by quantitative real-time PCR (qPCR) using sequence-specific primers (listed in Table 1). *Actb* (β-actin) was used as an internal control. qPCR experiments were conducted with Power SYBR Green PCR Master Mix (catalog no. 4367659, Thermo Fisher Scientific) by following the manufacturer’s protocol and real-time PCR system (7900HT, Applied Bioscience, Salt Lake City, UT, USA). Data analysis was performed by the ∆∆CT method.

### 4.8. Mitochondrial Membrane Potential Measurement

∆Ψm values were measured using 5,5′,6,6′ tetrachloro-1,19,3,39-tetraethylbenzimidazoyl-carbocyanine iodide (JC1, catalog no. T3168, Molecular Probes, Thermo Fisher Scientific, Waltham, MA, USA), a lipophilic cationic dye. Due to positive charges and lipophilic characteristics, JC-1 monomers concentrate to mitochondria where the membrane potentials are extremely negative (about −150 to −200 mV) and aggregate into polymers. While the monomers were excited at a wavelength of 490 nm and emitted 535 nm green fluorescence, the excitation and emission wavelengths of the polymers were 535 nm and 590 nm (red fluorescence), respectively. 

Brown adipocytes were seeded in 96-well black polystyrene microplates (catalog no. 3603, Corning, New York, NY, USA) a day before the experiment. The cells were incubated with 2 μM JC-1 in KRB solution for 30 min and then changed to T_3_ solution (in KRB) for 30 min before the measurement. The fluorescence signals were recorded by a fluorescence microplate reader (FlexStation II, Molecular Devices, San Jose, CA, USA). The ∆Ψm were estimated by a ratio of red/green (590 nm/535 nm).

### 4.9. Statistical Analysis

The statistical analysis was conducted using two-tailed unpaired Student’s *t*-test and one-way ANOVA (Tukey’s posthoc test) using GraphPad Prism 9.0 (GraphPad Software Inc, San Diego, CA, USA). Data were presented as mean ± SEM. *p* values below 0.05 were considered statistically significant.

## 5. Conclusions

In this study, we demonstrated that the acute exposure of brown adipocytes to T_3_ increases [Ca^2+^]_i_ via PLC activation. Elevated [Ca^2+^]_i_ increases mitochondrial respiration directly through mitochondrial Ca^2+^ uptake and metabolic activation and also through the increased abundance of mitochondrial proteins such as UCP-1. We suggest that [Ca^2+^]_i_ bound CaM activates mTOR signaling and inhibits autophagic degradation leading to increased mitochondrial protein abundance.

## Figures and Tables

**Table 1:** Primer sequences used for quantitative PCR.

Gene	Sequence
*Ucp1*	F-5′ TGGAAAGGGACGACCCCTAA 3′ R-3′CAAAACCCGGCAACAAGAGC 5′
*Ppargc1a*	F-5′ TGGAGTGACATAGAGTGTGCT 3′ R-3′ TTCCGATTGGTCGCTACACC 5′
*Pparg*	F-5′ ACCATGGTAATTTCAGTAAAGG 3′ R-3′ GTCTCGGTTGAGGGGACG 5′
*Prdm16*	F-5′ CGACTTTGGATGGGAGCAGAT 3′ R-3′ ACGGATGTACTTGAGCCAGC 5′
*Fndc5*	F-5′ CTCTTCATGTGGGCAGCTGTTA 3′ R-3′ GCGCTCTTGGTTTTCTCCTTG 5′
*Cd36*	F-5′ TTGAAAGCAGTGGTCCTTC 3′ R-3′ GCTGTTATTGGTGCAGTCCT 5′
*mTOR*	F-5′ CCGCCTTCACAGATACCCAG 3′ R-3′ TTAAACTCCGACCTCACGGC 5′
*Acaca*	F-5′ CCTGACAAACGAGTCTGGCT 3′ R-3′ CATTCCATGCAGTGGTCCCT 5′
*Acacb*	F-5′ GCCTGACCTTTTCCTGGCTA 3′ R-3′ TGAGGCTGAAAGGGACTCCT 5′
*Rps6*	F-5′GGAAGCCCTTAAACAAAGAAGGTAA 3′ R-3′ AATACGTCGGCGTTTGTGTT 5′
*Akt*	F-5′ GACTTCCGATCAGGCTCACC3′ R-3′ACTCGTTCATGGTCACACGG 5′
*Map1lca*	F-5′ CCAAGATCCCAGTGATTATAGAGC 3′ R-3′ TGCAAGCGCCGTCTGATTAT 5′
*Map1lcb*	F-5′ CCAAGATCCCAGTGATTATAGAGC 3′ R-3′ TGCAAGCGCCGTCTGATTAT 5′
*Actb*	F-5′ CGCTACAAGGGTGAGAAGCA3′ R-3′ GCGGCGCCGGATGAT 5′

## Data Availability

The data presented in this study are available in the published article and Supplementary Material.

## Supplementary Materials

The following are available online at https://www.mdpi.com/article/10.3390/ijms22168640/s1, Figure S1: T_3_ induces changes in functions of brown adipocytes’ mitochondria within 30 min. Figure S2: T_3_ induces changes in cell signaling within 30 min. Figure S3: T_3_-induced cytosolic Ca^2+^ increase regulates OCR and cell signaling alterations. Figure S4: Both [Ca^2+^]_i_-CaM activation and mitochondrial Ca^2+^ influx are involved in T_3_-induced OCR elevation. Figure S5: PLC inhibitors inhibit effects of T_3_ on brown adipocytes. Figure S6: T_3_ induces phosphorylations of AMP kinase and acetyl-coA carboxylase, Supplemental Methods.
