# Supplementary material for: Thyroid Hormone Induces Ca2+-Mediated Mitochondrial Activation in Brown Adipocytes
Source: Int J Mol Sci. 2021 Aug 11;22(16):8640. doi: 10.3390/ijms22168640 (PMC8395422; doi:10.3390/ijms22168640)
Supplement: Supplementary file 1 [file ijms-22-08640-s001.zip › ijms-1287383-supplementary.pdf]

International Journal of *Molecular Sciences*

Article

Thyroid Hormone Induces Ca^{2+}-mediated Mitochondrial Activation in Brown Adipocytes

Minh-Hanh Thi Nguyen [1,2,3], Dat Da Ly [1,2], Nhung Thi Nguyen[1,2], Xu-Feng Qi [4], Hyon-Seung Yi [5], Minho Shong [5], Seung-Kuy Cha [1,2,3], Sangkyu Park[1,6,*], Kyu-Sang Park [1,2,3,*]

[1] Mitohormesis Research Center, Yonsei University Wonju College of Medicine, Wonju, Korea; minh-hanh.tnguyen@yonsei.ac.kr (M.-H.T.N.); dadat.p10@gmail.com (D.D.L.); skcha@yonsei.ac.kr (S.-K.C.); skpark00@yonsei.ac.kr (S.P.); qsang@yonsei.ac.kr (K.-S.P.).
[2] Department of Physiology, Yonsei University Wonju College of Medicine, Wonju, Korea
[3] Department of Global Medical Science, Yonsei University Wonju College of Medicine, Wonju, Korea,
[4] Key Laboratory of Regenerative Medicine, Ministry of Education, Department of Developmental and Regenerative Biology, Jinan University, Guangzhou, China; qixufeng@jnu.edu.cn (X.-F.Q.)
[5] Department of Internal Medicine, Chungnam National University School of Medicine, Daejeon, Korea; jmpbooks00@gmail.com (H.-S.Y.); minhos@cnu.ac.kr (M.S.).
[6] Department of Precision Medicine, Yonsei University Wonju College of Medicine, Wonju, Korea
* Correspondence: skpark00@yonsei.ac.kr; Tel.: (+82-33-741-0154), qsang@yonsei.ac.kr; Tel.: (+82-33-741-0294)

Table of contents

Figure S1. *T_3 induces changes in functions of brown adipocytes' mitochondria within 30 min.*

Figure S2. *T_3 induces changes in cell signaling within 30 min.*

Figure S3. *T_3-induced cytosolic Ca^{2+} increase regulates OCR and cell signaling alterations.*

Figure S4. *Both $[Ca^{2+}]_i$-CaM activation and mitochondrial Ca^{2+} influx are involved in T_3-induced OCR elevation.*

Figure S5. *PLC inhibitors inhibit effects of T_3 on brown adipocytes.*

Figure S6. *T_3 induces phosphorylations of AMP kinase and acetyl-coA carboxylase.*

Supplemental Methods

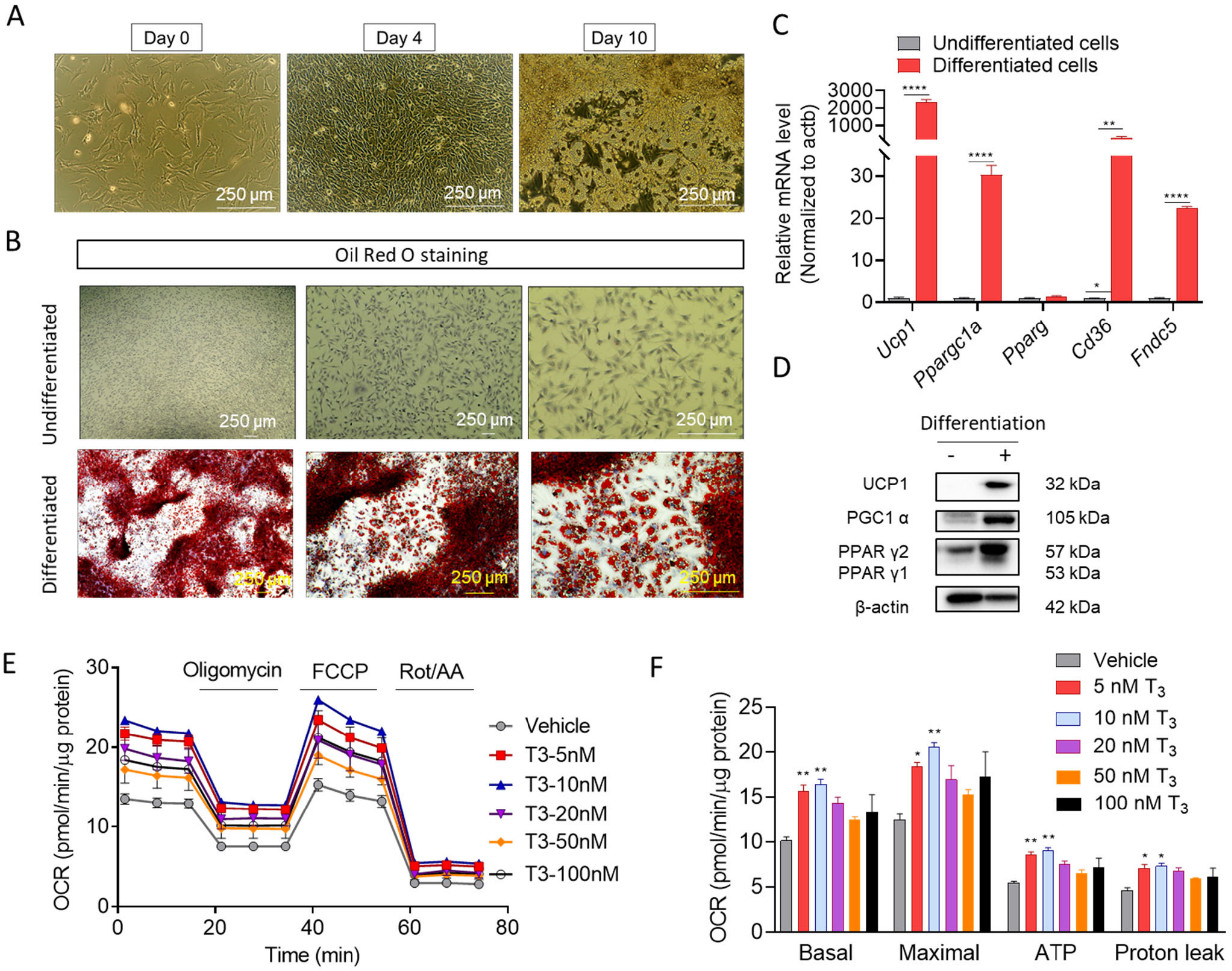

Figure S1. T_3 induces changes in brown adipocyte mitochondria functions within 30 min. (A) Immortalized brown adipocytes on day 0, 4, 10 of differentiation process. (B) Oil Red O staining of immortalized undifferentiated (upper) and differentiated (lower) brown adipocytes. (C) Relative mRNA levels of *Ucp1, Ppargc1a, Pparg, Cd36, Fndc5* (normalized to *actb*) (n=3) of immortalized brown adipocytes. (D) Western blot of UCP1, PGC1 α and PPAR γ on immortalized undifferentiated and differentiated brown adipocytes. (E) OCR measurement on immortalized brown adipocytes under the treatments of different concentrations of T_3 for 30 min. (F) Quantitative analysis of basal respiration, maximal respiration, ATP production, and proton leak (n=4). Data are presented as mean ± SEM. * $p \leq 0.05$, ** $p \leq 0.01$, **** $p < 0.0001$.

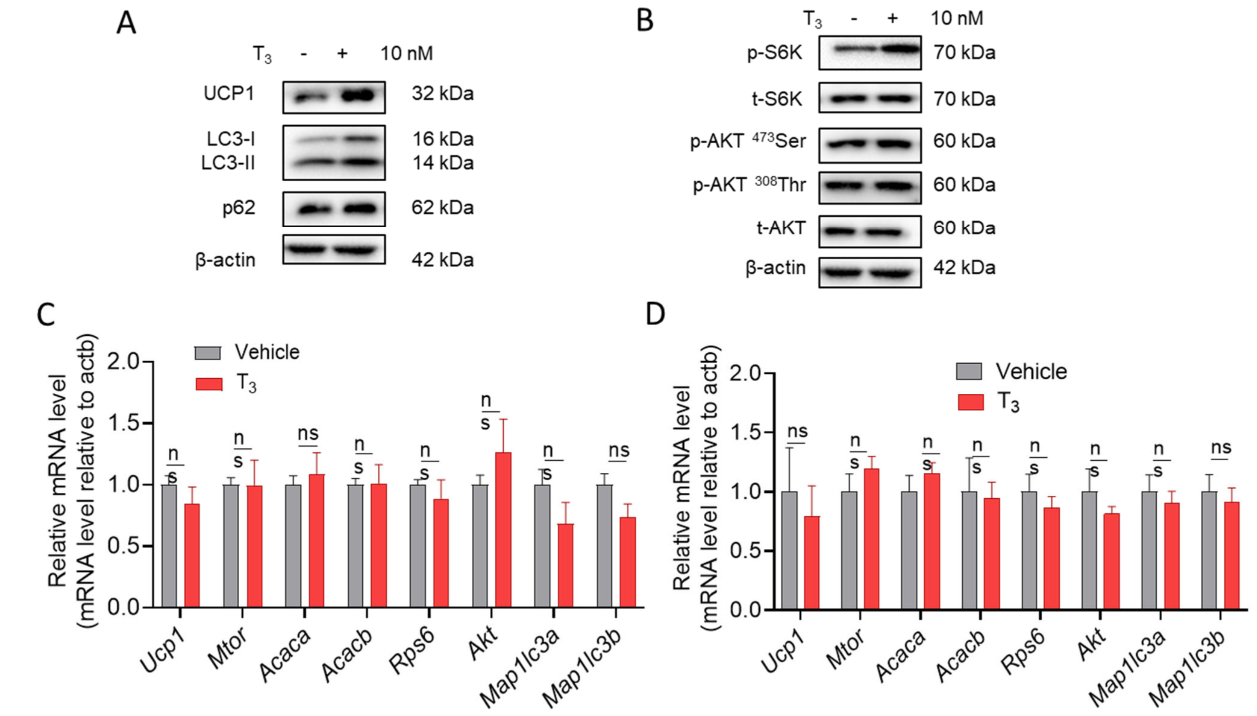

Figure S2. T_3 induces changes in cell signaling within 30 min. Western blots of (A) UCP1 and autophagy-related proteins, and (B) mTOR and akt signaling proteins with 10 nM T_3 treatment for 30 min in primary brown adipocytes. Relative mRNA levels of *Ucp1*, *Mtor*, *Acaca*, *Acacb*, *Rps6*, *Akt*, *Map1lca*, and *Map1lcb* with 30 min of T_3 treatment in (C) immortalized, and (D) primary brown adipocytes. Data are presented as mean ± SEM, ns not significant.

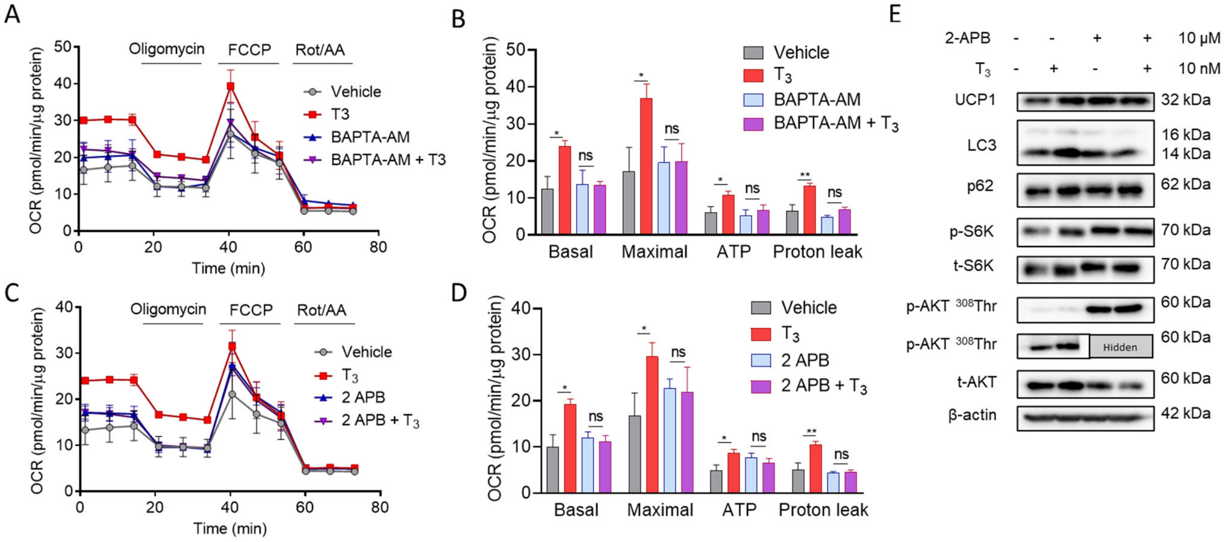

Figure S3. T_3-induced cytosolic Ca^{2+} increase regulates OCR and cell signaling alterations. (A) OCR measurement in immortalized brown adipocytes under 10 μM BAPTA-AM treatment for 1 h prior to 10 nM T_3 application for 30 min. (B) Quantitative analysis of basal respiration, maximal respiration, ATP production and proton leak. (C) OCR measurement in immortalized brown adipocytes under 10 μM 2-APB treatment for 1 h before 10 nM T_3 treatment for 30 min. (D) Quantitative analysis of basal respiration, maximal respiration, ATP production, and proton leak (n=4). (E) Western blot of UCP1, autophagy related proteins, and mTOR signaling proteins with 10 μM 2-APB treatment for 1 h before 10 nM T_3 treatment for 30 min in immortalized brown adipocytes. Data are presented as mean ± SEM. * $p \le 0.05$, ** $p \le 0.01$, ns not significant.

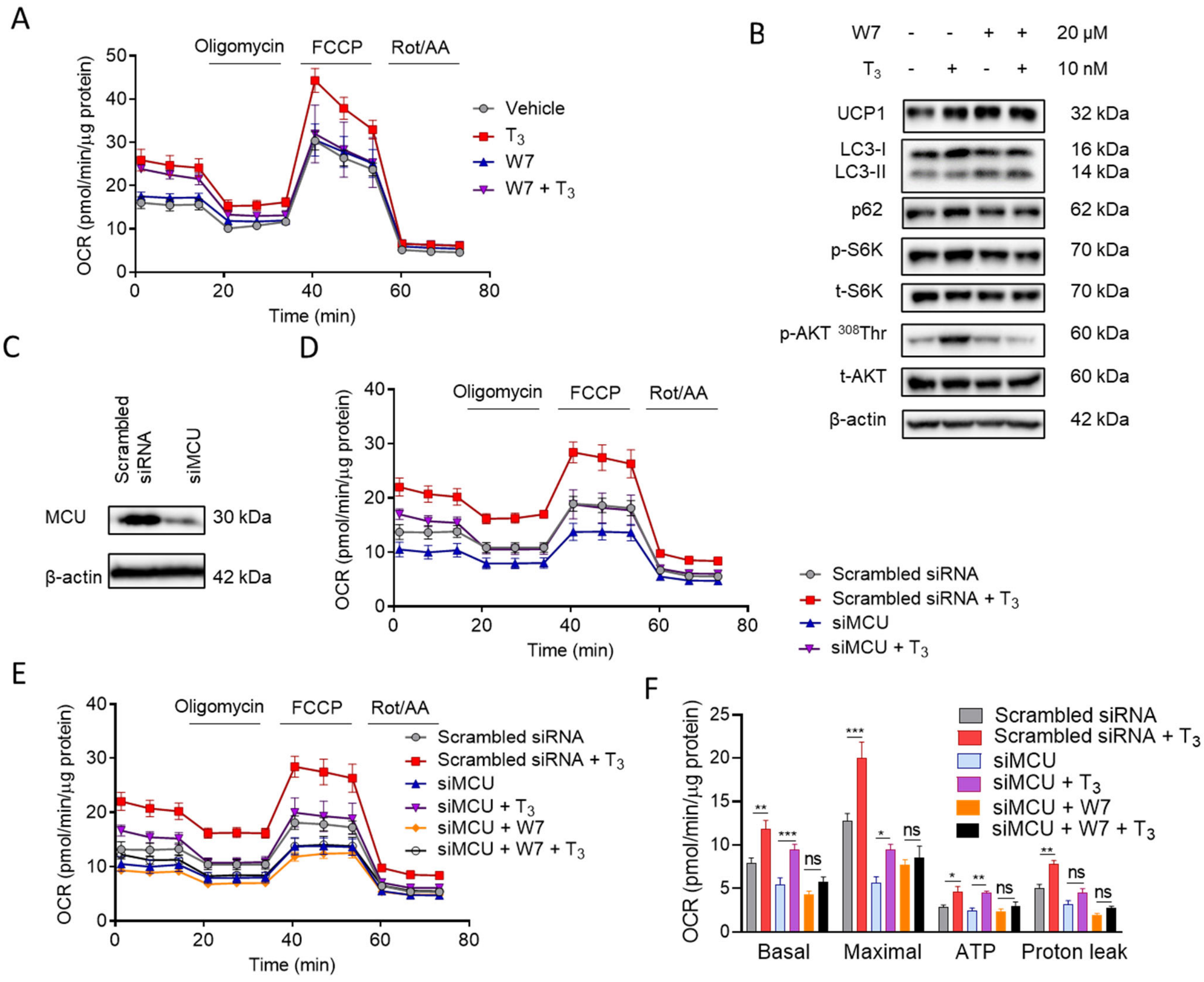

Figure S4. Both $[Ca^{2+}]_i$-CaM activation and mitochondrial Ca^{2+} influx are involved in T_3-induced OCR elevation. (A) OCR measurements in immortalized brown adipocytes with 20 µM W7 treatment for 1 h before T_3 treatment. (B)Western blot of several autophagy related proteins, and mTOR signaling pathway proteins in immortalized brown adipocytes with 20 µM W7 application for 1 hour before 10 nM T_3 treatment for 30 min. (C) Western blot of MCU under Scrambled siRNA and siMCU transfection in immortalized brown adipocytes. (D) OCR measurements of *Mcu* knockdown in immortalized brown adipocytes with 10 nM T_3 treatment for 30 min. (E) OCR measurements of *Mcu* knockdown immortalize brown adipocytes with 20 µM W7 treatment for 1 h before T_3 application. (F) Quantitative analysis of basal respiration, maximal respiration, ATP production, and proton leak. Data are presented as mean ± SEM. * $p \le 0.05$, ** $p \le 0.01$, *** < 0.001, ns not significant.

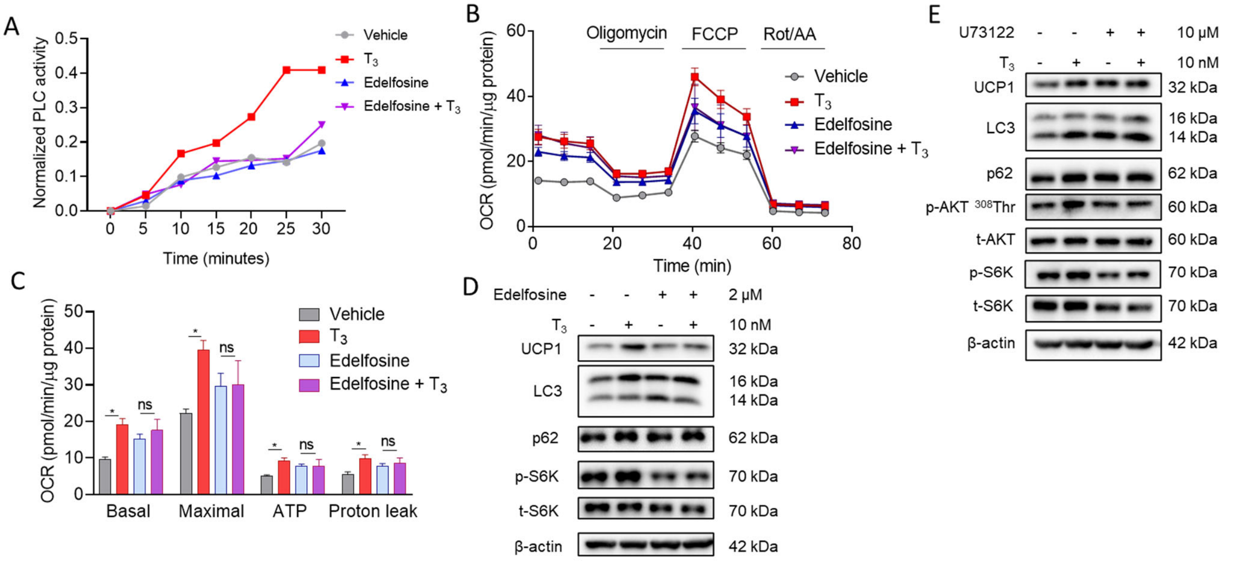

Figure S5. PLC inhibitors inhibit effects of T_3 in brown adipocytes. (A) PLC activity assay on immortalized undifferentiated brown adipocytes with 2 μM edelfosine treatment for 6 h prior 10 nM T_3 application for 30 min (B) OCR measurement in immortalized brown adipocytes with 2 μM edelfosine pretreatment. (C) Quantitative analysis of basal respiration, maximal respiration, ATP production and proton leak. (D)Western blot of UCP1, autophagy related proteins, and mTOR signaling proteins, with 2 μM edelfosine pretreatment in primary brown adipocytes. (E) Western blot of UCP1, autophagy related proteins, and mTOR signaling proteins, with 10 μM U73122 treatment for 6 h before 10 nM T_3 treatment for 30 min in immortalized brown adipocytes. Data are presented as mean ± SEM. * $p \le 0.05$, ns not significant.

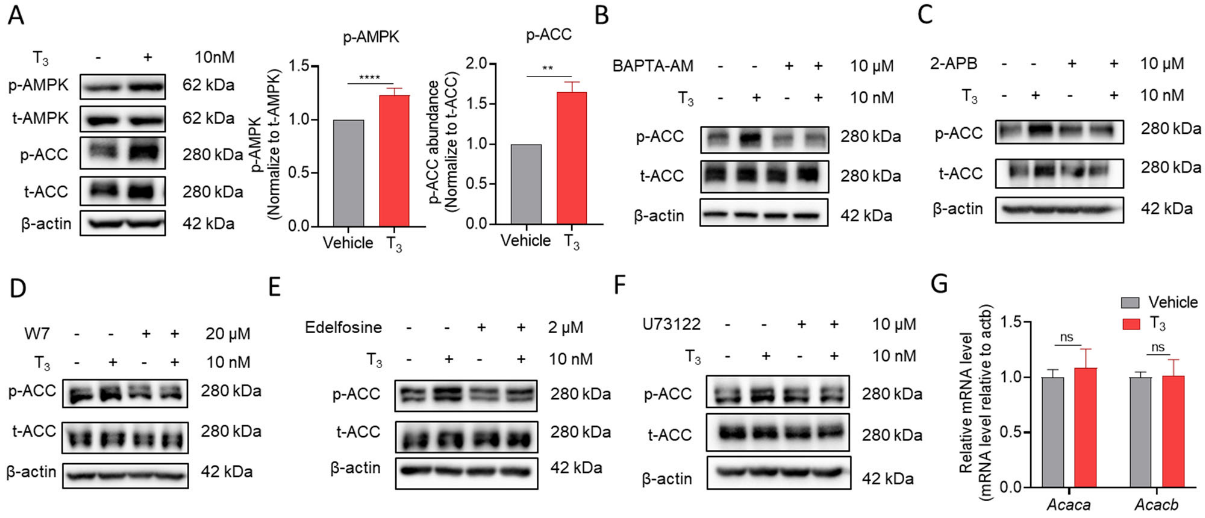

Figure S6. T_3 induces phosphorylations of AMP kinase (AMPK) and acetyl-coA carboxylase (ACC). (A) Western blot of AMPK and ACC with 10 nM T_3 treatment for 30 min in immortalized brown adipocytes. (B) Western blot of ACC in immortalized brown adipocytes with 10 μM BAPTA-AM treatment for 1 hour before 10 nM T_3 treatment for 30 min. (C) Western blot ACC with 10 μM 2-APB treatment for 1 h before 10 nM T_3 treatment for 30 min in immortalized brown adipocytes. (D) Western blot of ACC in immortalized brown adipocytes with 20 μM W7 treatment for 1 hour before 10 nM T_3 treatment for 30 min.(E) Western blot of ACC with 2 μM edelfosine treatment for 6 h before 10 nM T_3 treatment for 30 min in immortalized brown adipocytes. (F) Western blot of ACC with 10 μM U73122 treatment for 6 h before 10 nM T_3 treatment for 30 min in immortalized brown adipocytes. (G) Relative mRNA levels of *Acaca,* and *Acacb* with 30 min of T_3 in immortalized brown adipocytes. Data are presented as mean ± SEM. ** $p \le 0.01$, **** $p < 0.0001$, ns not significant.

Supplemental Methods

1. Small interfering RNA transfection

Brown adipocytes were transfected with 30 nM SiGENOME Smart-pool small interfering RNA (siRNA) duplexes for mouse *Mcu* (siGENOME; catalog no. 062849-01, Dharmacon, Thermo Fisher Scientific, Waltham, MA, USA) or negative control (catalog no. SN-1002, Bioneer, Daejeon, South Korea) mixed with DharmaFECT siRNA transfection reagent (catalog no. T-2001-03, Thermo Fisher Scientific, Waltham, MA, USA). The transfection efficacy was assessed after 72 h by western blotting.

2. PLC activity assay

Immortalized undifferentiated brown adipocytes were treated with 2 μM edelfosine for 6 h prior 10 nM T_3 application for 30 min. The PLC activity was measured with Phospholipase C activity assay kit (catalog no. ab273343, Abcam, Cambridge, UK), based on manufacturer's instruction. The cells were lysed with PLC Assay buffer and then centrifuged at 10,000 x g at 4 °C for 20 min to collect the supernatants. The supernatants were mixed with PLC Assay buffer and reaction mix following the manufacture's protocol and incubated at 37 °C along with standard curve. The reaction product was measured the OD at 405 nm every 5 min. The data was normalized using the formulation equation (1).

$$Normalized\ PLC\ activity = \frac{A_t - A_0}{A_0} \quad (1)$$

A_t, A_0: Amount of colorimetric product generated from the Standard Curve (nmol) at 0 (min) or t (min) respectively.
